# Microstructural Investigation of a Friction-Welded 316L Stainless Steel with Ultrafine-Grained Structure Obtained by Hydrostatic Extrusion

**DOI:** 10.3390/ma14061537

**Published:** 2021-03-21

**Authors:** Beata Skowrońska, Tomasz Chmielewski, Mariusz Kulczyk, Jacek Skiba, Sylwia Przybysz

**Affiliations:** 1 Faculty of Production Engineering, Warsaw University of Technology, Narbutta 85, 02-524 Warsaw, Poland; tomasz.chmielewski@pw.edu.pl; 2 Institute of High Pressure Physics, Polish Academy of Sciences (Unipress), Sokołowska 29, 01-142 Warsaw, Poland; mariusz.kulczyk@unipress.waw.pl (M.K.); jacek.skiba@unipress.waw.pl (J.S.); sylwia@unipress.waw.pl (S.P.)

**Keywords:** 316L stainless steel, hydrostatic extrusion, rotary friction welding, microstructure, ultrafine-grained metal, recrystallization

## Abstract

The paper presents the microstructural investigation of a friction-welded joint made of 316L stainless steel with an ultrafine-grained structure obtained by hydrostatic extrusion (HE). Such a plastically deformed material is characterized by a metastable state of energy equilibrium, increasing, among others, its sensitivity to high temperatures. This feature makes it difficult to weld ultra-fine-grained metals without losing their high mechanical properties. The use of high-speed friction welding and a friction time of <1 s reduced the scale of the weakening of the friction joint in relation to result obtained in conventional rotary friction welding. The study of changes in the microstructure of individual zones of the friction joint was carried out on an optical microscope (OM), scanning electron microscope (SEM), transmission electron microscope (TEM) and electron backscattered diffraction (EBSD) analysis system. The correlation between the microstructure and hardness of the friction joint is also presented. The heat released during the high-speed friction welding initiated the process of dynamic recrystallization (DRX) of single grains in the heat-affected zone (HAZ). The additional occurrence of strong plastic deformations (in HAZ) during flash formation and internal friction (in the friction weld and high-temperature HAZ) contributed to the formation of a highly deformed microstructure with numerous sub-grains. The zones with a microstructure other than the base material were characterized by lower hardness. Due to the complexity of the microstructure and its multifactorial impact on the properties of the friction-welded joint, strength should be the criterion for assessing the properties of the joint.

## 1. Introduction

The basic criteria determining the suitability of a material for a particular construction are, among others, its rigidity (represented by an appropriate modulus of elasticity) and strength (interpreted in different ways depending on the type of material). It has been well known that strength and toughness of polycrystalline metals are improved with decreasing the grain size. Concerning the strength, Hall-Petch relationship was empirically described in the formula (1).

σ_y_ = σ_0_ + kd^(−1/2)^ (1)

where: σ_y_ is the yield strength of the polycrystalline material, σ_0_ the friction stress, k the Hall-Petch constant, and d the average grain size [1].

Significant refinement of the microstructure can be achieved through, among others adding more alloying elements and/or applying complex thermomechanical treatments [2]. However, they are costly methods and often their potential for improvement is limited. The solution to the limitations of classic methods of metal strengthening is, e.g., cold plastic processing of metal or more effective metal forming processes with severe plastic deformation (SPD) [3] In these processes, the increase in mechanical properties is achieved by controlled grain refinement at low temperatures [4,5]. The SPD process allows for obtaining high grain refinement to a nanometric level (where the average grain size is below 100 nm). Also, the refinement of grains above 100 nm but below 1 µm (ultrafine-grained materials) causes a significant increase in mechanical properties [6,7]. And the use of such materials as construction materials allows to significantly reduce the weight of the construction or reduce the quantity of expensive elements used as alloying additives.

One of the methods that enables strong grain refinement is the hydrostatic extrusion—a plastic deformation process that differs from conventional direct extrusion by the presence of a liquid medium (usually oil) [8]. The results of Sillekens’ research [9] comparing, conventional extrusion with hydrostatic has shown that HE eliminates the disadvantages of the conventional method such as heterogeneity in the degree of deformation and properties of the extruded elements, as well as the formation of a texture gradient (due to process conditions). It has also been observed that the frictional forces occurring during HE are incomparably lower, which significantly lowers the pressures needed for plastic working and there is a smaller temperature increase during hydroextrusion [10,11]. It is i.a. the effect of the three-axis stress state created during the HE process by the medium surrounding the extruded material in the working chamber. Depending on the type of material and the obtained degree of microstructure refinement, the hydrostatic extrusion process is carried out once or several times (resulting in high cumulative deformation). The application of HE to materials, i.e., Al, Al-Si, Ag and Cu [12], silicon bronze (C65500) [13], Ti [14], Ni [15] resulted in, in all cases, an increase in strength of properties. The influence of the hydrostatic extrusion process on changes in the microstructure, mechanical and physical properties in 316L austenitic steel is described in [16,17].

In order to determine the sensitivity to welding (high temperature thermal cycle) of materials with fine grained microstructure obtained by means of plastic deformation, knowledge of the chemical composition and basic mechanical properties is not sufficient. Because the course of the process, i.e., thermomechanical rolling or SPD, also affects the internal energy of the deformed material [18,19]. During cold plastic deformation of polycrystalline materials, some of the energy not converted into heat is stored inside the material (defragmented grains). It mainly accumulates in regions around lattice defects (i.e., dislocations, twins). The increase in free energy over the energy corresponding to the equilibrium state of the deformed material (annealed and slowly cooled) causes that it remains in the metastable equilibrium state (stable at ambient temperature). Therefore, the stored energy of the material significantly affects its thermal stability and sensitivity to welding. Since materials with a greater deviation from equilibrium according to the laws of thermodynamics, they will need a smaller stimulus, e.g., in the form of a thermal pulse at a lower temperature/energy, to bring them back to equilibrium.

Regardless of the fusion welding method used, energy is supplied to the material in the form of heat to create a joint. Stored strain energy is also a driving force for processes such as recovery and recrystallization [20]. They cause structural changes that bring the material closer to the state of thermodynamic equilibrium, and the energy stored is gradually released. Although, conventionally recrystallization phenomenon happening during annealing after plastic deformation is the major way of increasing the grain size of metallic materials [21], in the case of semi-finished products with the e.g., Ultrafine-Grained (UFG) structure it is an unfavorable phenomenon. It has been observed that in materials subjected to thermomechanical treatments or deformed by means of severe plastic deformation process, the phenomenon of recrystallization causes the loss of high mechanical properties (refinement of the UFG structure) [22]. Therefore, welding of such materials without losing their high properties, especially in the heat-affected zone, is much more difficult. For joining thermomechanically processed steels with a thickness of 10 mm, the use of hybrid welding methods, i.e., laser + metal active gas (MAG) [23] or plasma + MAG [24,25], reduced the amount of heat supplied to the material and limited the range of recrystallization. Also, the weld formed by melting and primer crystallization of the parent material during laser welding was characterized by lower mechanical properties (i.e., hardness and impact toughness) than, for example, a MAG weld (in which, together with the additional material, elements increasing the mechanical properties of the weld are supplied) [26]. Positive effect of arc welding of thermomechanical steels in a water environment on reducing structural degradation in HAZ was also observed [27,28].

The way to reduce the degradation of the microstructure of the ultrafine-grained material can also be the use of one of the methods of the solid-state joining process, such as: rotary friction welding (RFW), linear friction welding (LFW), friction stir welding (FSW) [29,30], and diffusion bonding [31]. These methods are often used to join materials that are difficult to welding [32,33] or have significantly different physical and mechanical properties [34,35,36]. The undoubted advantage of the solid-state joining process in terms of joining highly deformed materials is that during friction welding of the same materials, the process takes place below its melting point. Despite the advantages of using materials with a fine microstructure and solid-state joining methods, a few publications describe friction welding of UFG and most of them concern joining metals and their alloys with quite high plasticity [37,38]. However, the described results confirm the possibility of obtaining (with appropriately selected parameters) limiting the degradation of the microstructure and the loss of increased properties of the native material in the heat-affected zone.

Joining process at weldability of UFG 316L steel after hydrostatic extrusion by means of classic rotary friction welding was not widely used and reported in literature (because of high risk of degradation in HAZ), but the high-speed friction welding (HSFW) method was used in [39,40].

The main advantage of that approach is the possibility of shortening time of welding cycle and narrowing the HAZ. Due to these parameters, during HSFW a high temperature gradient is obtained. Additionally, occurring of high temperature is on friction surface mainly and risk of overheating in HAZ is relatively low.

This paper presents an analysis of changes in the microstructure of individual zones of joints caused by the high-speed friction welding process. Additionally, these changes were correlated with the results of hardness measurements in welded joints.

## 2. Materials and Methods

**Figure 1:**
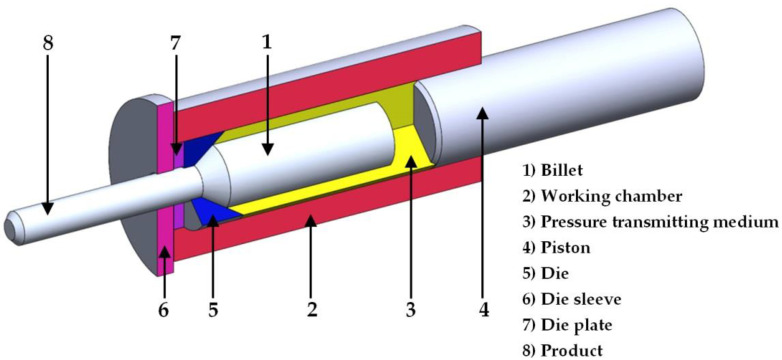
Scheme of the process of hydrostatic extrusion.

The material subjected to high-speed friction welding was 316L austenitic steel after the hydrostatic extrusion process. The principle of the HE method is presented in Figure 1. The billet (1) is placed in a working chamber (2), the remaining chamber space is filled with the pressure transmitting medium (3). The moving piston (4) compresses the medium, increasing the hydrostatic pressure acting on the billet. After obtaining a critical pressure value (characteristic for the extruded material) and after overcoming the frictional resistance of the charge-die, the process of plastic deformation begins. The material is pressed through the hole in the die (5) sliding on the layer made of the working liquid and the lubricant applied to the billet (before the HE process). The extruded product (8) is cooled just behind the die. Depending on the die used, different shapes of profiles and pipes cross-sections, as well as a degree of reduction ratio (*R*) and true strain (*ε*) are obtained. True strain in the HE process depends on the ratio of the cross-section before and after the extrusion process and is calculated according to formula [41]:

(2) $$\varepsilon =lnR=ln\left(\frac{A_{o}}{A_{f}}\right)=ln\left(\frac{d_{o}^{2}}{d_{f}^{2}}\right)\;$$

where: *R* is the degree of reduction, *A_o_* and *d_o_*—cross-section or diameter of material before extrusion, *A_f_* and *d_f_*—cross-section or diameter of the material after extrusion.

In paper [17], the hydrostatic extrusion of 316L austenitic steel was characterized with the use of the reduction ratio in the range of 1.6 < *R* <4.85, the corresponding true strain of 0.47 < *ε* <1.58 and their impact on changes in the microstructure and mechanical properties of the material. The friction-welded rods were hydrostatically extruded in one pass with *R* = 3.42, which corresponds to *ε* = 1.23. As a result, a rod with a diameter of 6 mm was obtained, its chemical composition and mechanical properties are presented in Table 1.

**Figure 2:**
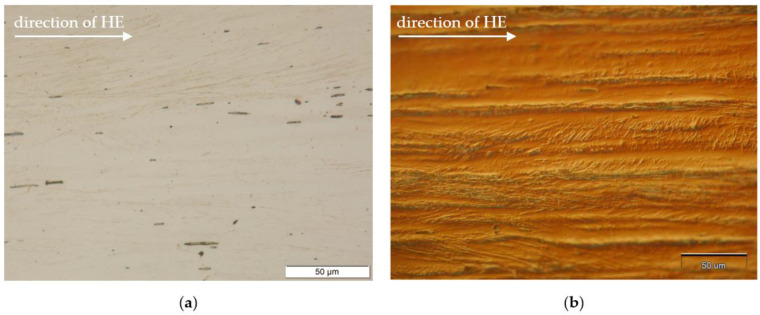
Micrograph of the microstructure of the base material after one-pass HE, longitudinal cross-section, (**a**) optical observation (**b**) with Nomar—Differential Interference Contrast (DIC).

The use of HE resulted in the strong modification of the typical 316L rod microstructure after metallurgical processes—the microstructure with equiaxed grains (and annealing twins) and the formation of a strongly band microstructure. Figure 2 shows microstructure of the native material (after HE), observed in the direction of rod extrusion, because further analysis of welded joints had been realized in the same plane. The application of the Nomar System—Differential Interference Contrast (DIC) revealed the topography of the band microstructure (Figure 2b).

The preparation of the 316L steel bars for welding after the HE, consisted of cutting into sections with a length of 26 mm, in order to avoid too much heat causing changes in the microstructure of the material, the cutting was made on a band saw with the use of cooling and emulsion lubrication.

Friction-welded joints made of 316L austenitic steel after hydrostatic extrusion were made on the welding machine Harms Wende HWM RSM200 achieving rotational speeds above 6000 rpm. In order to select the best set of parameters, numerous quick friction welding tests were carried out. For friction-welded joints, it is most desirable that the heat-affected zone (HAZ) has an even distribution (the same width) along the resulting friction weld. From the friction welding tests carried out with various sets of parameters, the best joint shape and the highest strength at the level of UTS = 1080 MPa and YS = 1017 MPa (Table 1) showed the joint made with the parameters presented in Table 2. Therefore, this friction-welded joint was selected for detailed microstructure tests.

Frictionally welded joints were tested on the cross-section (along the axis of the bars). For this purpose, the samples were first cut on wire electrical discharge machining (WEDM)—the cutting line was 0.5 mm away from the rod axis to leave an allowance for grinding and polishing operations. Samples for OM, SEM and hardness measurements after inclusion in epoxy resin were wet grinding with the use of discs with a grain size of 80 to 2500. After obtaining the axis of the bars, polishing was performed on a polishing cloth with Al_2_O_3_ suspension. Finally, the samples were etched with Mi16Fe (10 mL HNO_3_ + 30 mL C_3_H_8_O_3_ + 20 mL HF) reagent. Samples for TEM-investigation were taken in the axis of the rods in a cross-section parallel to the axis and perpendicular to the friction weld. Thin foils were prepared from disc-shaped samples 3 mm in diameter and about 0.15 mm thick. The discs were pre-ground with 1000 grit paper and electrolytically polished using the TenuPol—5STRUERS machine. Preparation of samples for EBSD-investigation included grinding of samples on silicon carbide abrasive papers of decreasing granulation (from 400 to 4000). The last stage was polishing with the use of a diamond suspension with a grain size of 3 and 0.25 µm and a SiO_2_ suspension with a grain size of 0.1 µm. The metallographic observations of the friction-welded joint were made under optical microscope Olympus BX51M (equipped with differential interference contrast), coupled with a digital camera and a computer with Olympus Stream Essentials software, with magnification from 25× to 500×. The microstructure was observed on a JEOL JSM-7600F scanning electron microscope (SEM) equipped with Schottky field emission gun (FEG), a JEOL JEM 1200 EX transmission electron microscope (TEM) and a Quanta 3D FEG high-resolution SEM equipped with e.g., electron backscattered diffraction (EBSD) analysis system.

Measurements of the hardness distribution across the friction-welded joint were carried out using the Vickers method on a LEITZ MINILOAD 8375 hardness tester at a load of 100 g and a 15 s test period. The method can be used for both hard and soft materials.

## 3. Results

### 3.1. The Optical Metallography of Friction-Welded Joint

**Figure 3:**
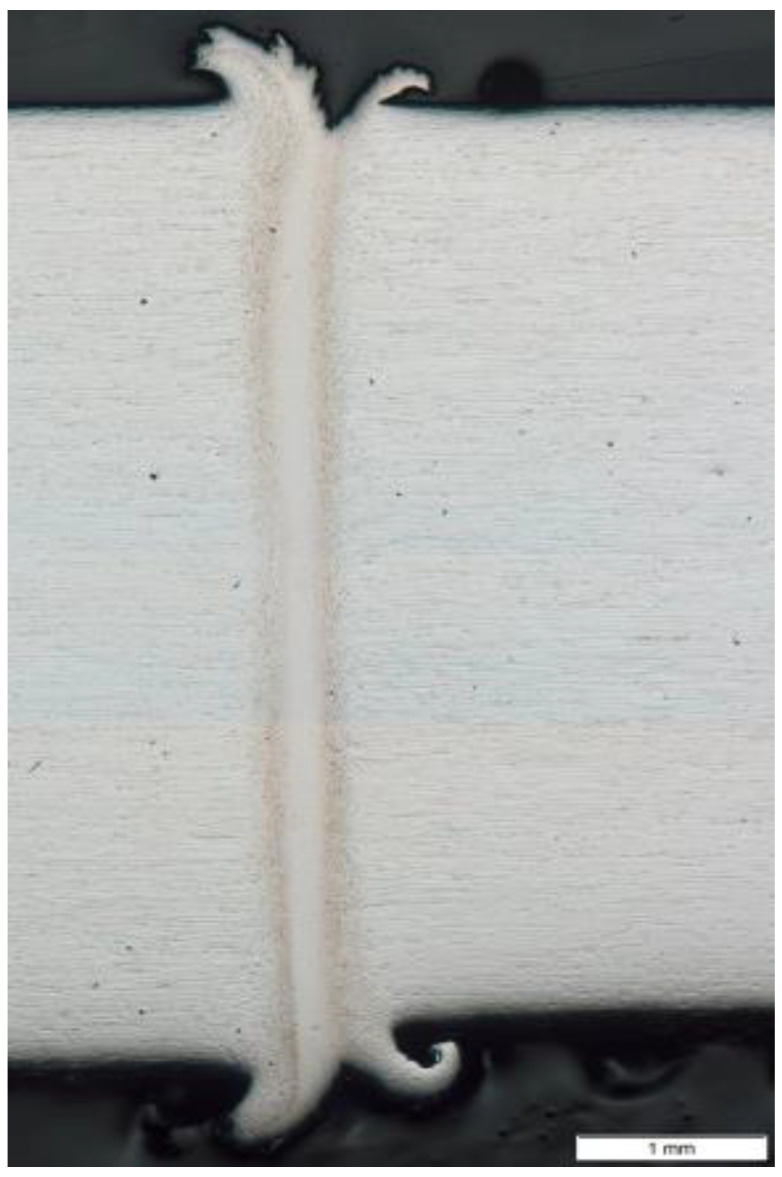
Macrostructure of the friction-welded joint from 316L steel after HE.

Macroscopic observations of the analyzed friction-welded joint (Figure 3) showed the previously mentioned uniform distribution of HAZ along the friction weld and no defects at the macro level. The friction-welded joint was characterized by a narrow heat-affected zone with a width of approx. 0.6 mm and a friction weld width of less than 0.2 mm.

**Figure 4:**
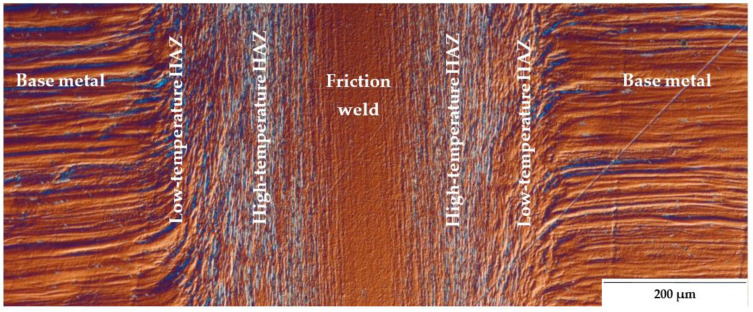
Microstructure of the friction joint from 316L steel after HE with Nomarski—Differential Interference Contrast (DIC).

Microstructure studies have revealed that for UFG 316L steel friction-welded joints, the heat-affected zone (HAZ) has a dual character of microstructure. Therefore, in Figure 4, an additional division of the HAZ was made into high- and low-temperature HAZ. The low-temperature HAZ is a zone of partial plasticized material, where the bending of its base material bands in the radial direction is visible. The high-temperature HAZ is characterized by a vertical arrangement of the strands along the friction weld, in this zone the material is fully plasticized. The weld is the area where the streak microstructure of the base material has been completely lost. The observations of the microstructure of the joint with DIC showed that the weld zone, in comparison with the others, has the most homogeneous character.

Assuming that the phenomena occurring during rotary friction welding are symmetrical. In the case of homonymous joints on both sides of the weld, the same effect of changes in the microstructure is obtained (Figure 4), therefore further characteristics of the observed zones of the friction-welded joint have been presented for one side of the joint only.

### 3.2. SEM Investigation of Welded Joints

**Figure 5:**
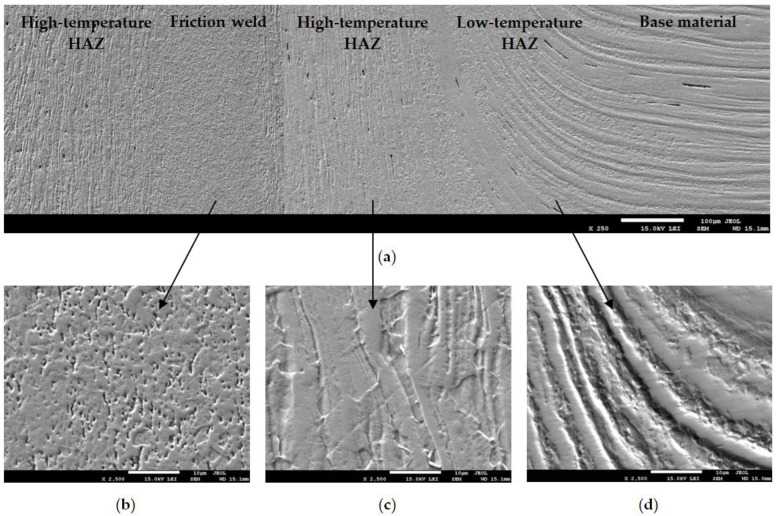
Microstructure of the transverse cross-section of friction-welded joint of UFG 316L steel. SEM-observation in the LEI mode: (**a**) mag. ×250, (**b**–**d**) mag. ×2500, of friction weld, high- and low-temperature HAZ, respectively.

The SEM-investigation using the lower electron image (LEI) detector was carried out to more accurately illustrate the changes in the microstructure of the material after hydrostatic extrusion caused by the high-speed friction welding process. Figure 5a shows a cross-section (at the axis of the rods) of a fragment of friction-welded joint, while Figure 5b–d show the microstructures of individual zones made at 10 times magnification than in Figure 5a, with magnification 2500×.

The HAZ, in which the lower temperature occurs, is characterized by strongly bent strands in the radial direction (up to the flash). Figure 5d also shows a strong difference in topography of the individual strands. A different configuration of the microstructure was shown by the high-temperature HAZ (Figure 5c). The friction welding process did not cause the loss of the bands, but a change in their arrangement from horizontal to vertical, along the frictional weld. Additionally, the vertical strands are disturbed by the lines that are most likely the grain boundaries. In the friction weld, the streaked character of the base material was completely lost (Figure 5a). In the presented friction-welded joint, it is not possible to distinguish interface. The weld shows a homogeneous microstructure with evenly distributed micro-voids.

The SEM-investigation also showed a different shape and frequency of micro discontinuities in individual zones of the friction-welded joint (Figure 5a). Horizontal micro discontinuities were observed in the base material, their distribution was random. In the HAZ they change their position according to the arrangement of the bent or vertical material strands. Due to the friction occurring on the front surfaces of the rods to be joined, the micro discontinuities in the friction weld have a completely different character than in other areas of the friction joint.

### 3.3. TEM Investigation of Friction-Welded Joint

**Figure 6:**
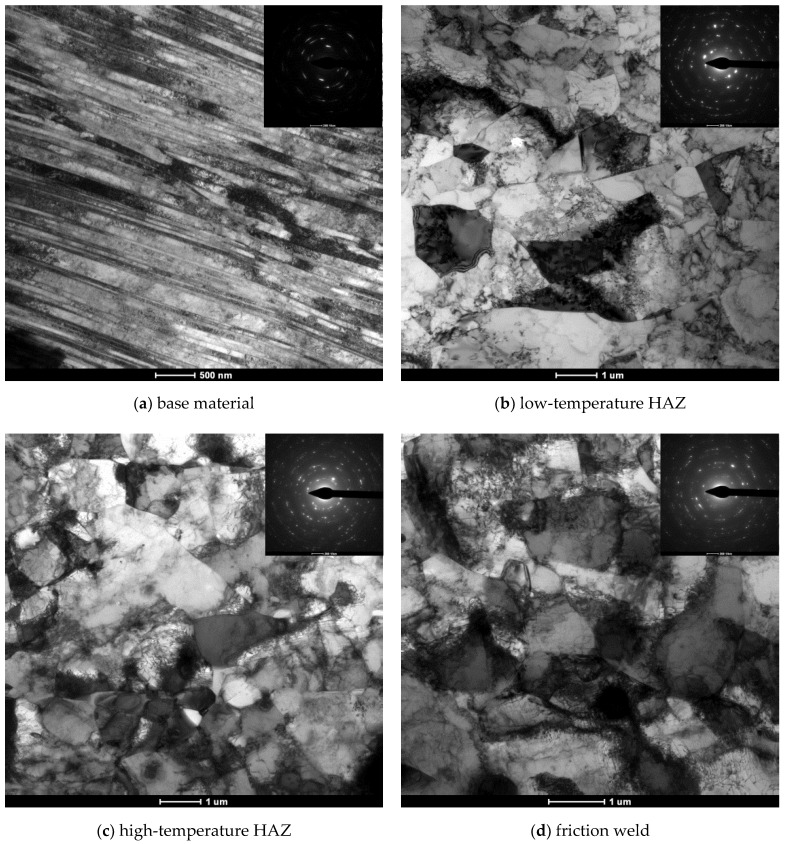
Bright field TEM images with corresponding SAED patterns obtained for (**a**) base material in longitudinal section to HE, (**b**) low-temperature HAZ, (**c**) high-temperature HAZ, (**d**) friction weld in transverse cross-section of the friction-welded joint.

The thin foils for TEM investigation were prepared from a transverse cross-section of the friction-welded joint. Perforations were made in two areas, in the HAZ between the high- and low-temperature zones and between the friction weld and the high-temperature HAZ. A sample for testing the base material after hydrostatic extrusion was prepared separately. Figure 6 shows the TEM images in a bright field with the corresponding selected area electron diffraction (SAED) patterns obtained for each area of a friction-welded joint.

The use of hydrostatic extrusion to plastically-deformed the 316L steel resulted in the formation of a strands structure in the extrusion direction. The width of individual strands is below 500 nm (Figure 6a), while it is not possible to observe the boundaries of individual grains.

In the heat-affected zone (both low- and high-temperature, Figure 6b,c), it can be observed that the conditions occurring during high-speed friction welding, despite the friction phase of only 60 ms, caused the phenomenon of dynamic recrystallization. Among the highly damaged microstructure with numerous dislocation weaves, in which it is impossible to distinguish the grains and sometimes also the sub-grains, the boundaries of single recrystallized grains can also be observed. Friction weld (Figure 6d) is characterized by strongly defective grains full of dislocation tangles both close to the boundaries and inside the grains themselves.

### 3.4. EBSD Investigation of Friction-Welded Joint

**Figure 7:**
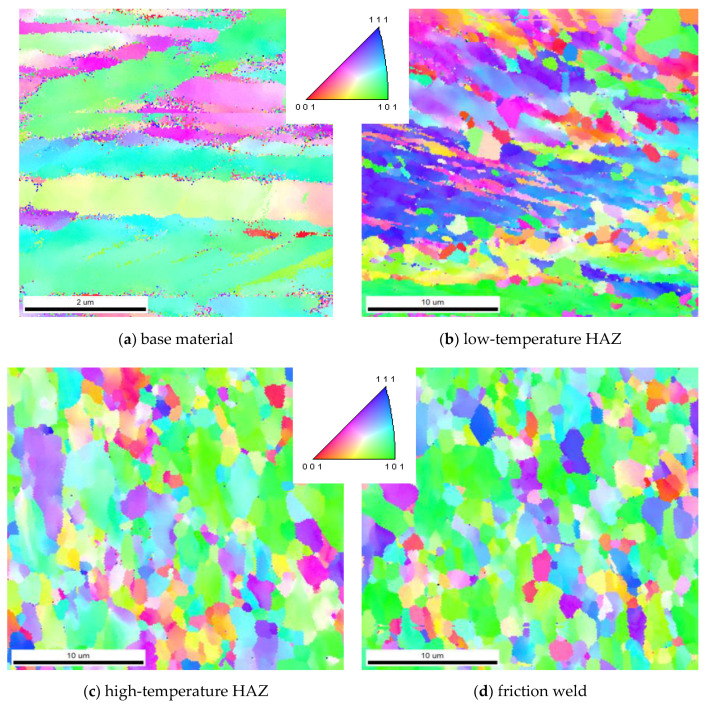
Inverse pole figure (IPF) maps for different zones of the friction-welded joint.

**Figure 8:**
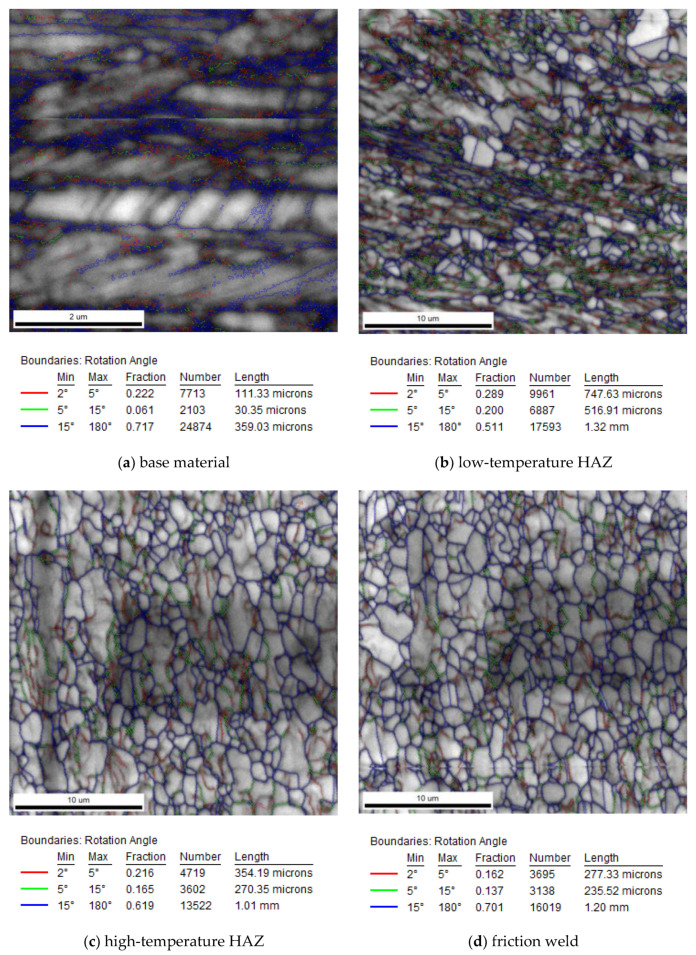
The micropatterns of the misorientation grain boundaries for different areas of the weld (corresponding to IPF maps).

**Figure 9:**
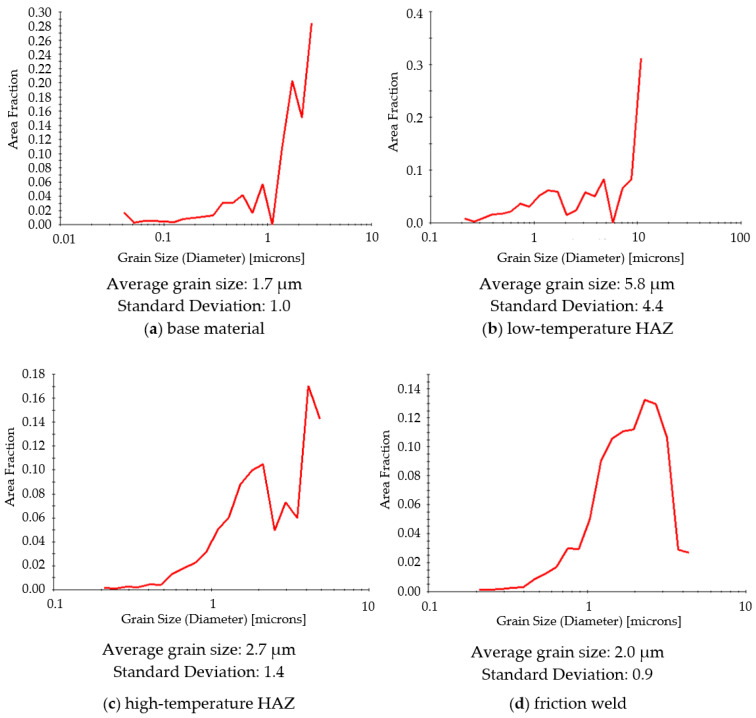
Grain size distribution in characteristic zones of friction-welded joints.

**Figure 10:**
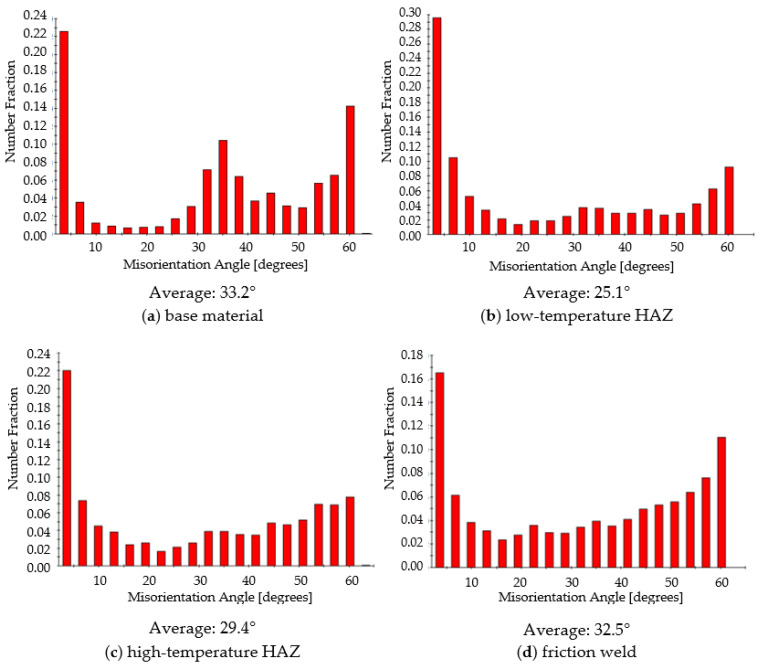
Distribution of misorientation angles in individual areas of the friction joint (corresponding to the IPF maps in Figure 7).

The microstructure of the characteristic zones of the friction-welded joint (marked in Figure 4) was also tested by EBSD. The tests were performed at the same height—at a distance of 1/4 of the radius from the edge of the rods, without taking into account the height of the resulting flash. The maps from the EBSD analysis showing the following microscopic information are presented below: grain orientation map and grains boundary map. For the grain orientation maps (Figure 7), the inverse pole figure (IPF) was used, showing the three main crystallographic directions in grains distinguished by different colors; red for (001), green for (101) and blue for (111). Figure 8 shows the maps for the distribution of grain boundaries in the microstructures of the studied areas. The low-angle grain boundaries with misorientation degree of 2°–5° were highlighted in red, the low-angle grain boundaries (LAGBs) with 5°–15° in green and the high-angle grain boundaries (HAGBs) with misorientation degree larger than 15° were marked in blue. The distributions of grain size and misorientation angles in individual areas of the friction-welded joint, along with the average values, are summarized and presented in Figure 9 and Figure 10, respectively.

Grain orientation and grains boundary maps confirmed the different nature of changes in the microstructure in the various zones of the friction-welded joint.

It was observed that the base material (316L steel after hydrostatic extrusion) is characterized by significantly plastically deformed crystalline microstructure of band character with numerous dislocation tangles and sub-grains (Figure 7a), and the fraction of HAGBs was 72% (Figure 10a). The average grain size was 1.7 µm, with the largest share of grains below 1 µm (25.5%) and numerous grains below 100 nm (Figure 9a). In the heat-affected zone, not only a significant change in microstructure can be observed compared to the base material, but also large differences in the size and arrangement of grains in the separated low- and high-temperature HAZ. In the low-temperature HAZ (Figure 7b), single recrystallized grains can be observed among the deformed grains arranged in the direction of rod extrusion and bending of the strands. The average grain size was 5.8 µm (Figure 9b). In this zone, the length of the grain boundaries with low- and low-angle misorientation was comparable to the high-angle ones (Figure 8b). In the high-temperature HAZ (Figure 7c), and also in the friction weld (Figure 7d), grains partially stretched in the vertical direction, surrounded by numerous equiaxial fine grains, were observed. The average grain size was 2.7 µm in the HAZ (Figure 9c) and 2 µm in the friction weld (Figure 9d), with almost 50% of the surface of the grains in the friction weld being in the range of 1–2 µm. The friction weld is characterized by the most uniform microstructure. In both cases, most of the grain boundaries are HAGBs (Figure 8c,d and Figure 10c,d).

For all tested zones of the friction-welded joint, in the misorientation angles distributions there is a high peak for the angle of 3° (Figure 10), which is typical for the material after SPD treatment [42]. However, the misorientation angle size for all of them is of the high angle type (above 15°). The base material and friction weld are characterized by grain boundaries with the highest mean value of the disorientation angle (approx. 33°).

### 3.5. Hardness Measurements

Due to the results of microscopic observations and the differences observed between the analyzed zones of the friction-welded joint, measurements of microhardness across the joint were also made. The measurement points, apart from the previously tested zones (i.e., the weld, high- and low-temperature HAZ and native material), were also planned in the areas in the transition areas between these zones. The measurement was taken near the longitudinal axis of the rod/joint.

**Figure 11:**
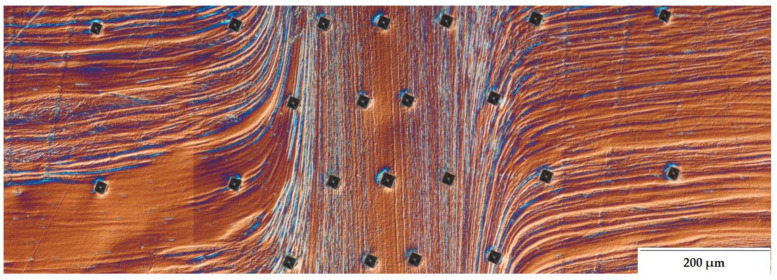
Image showing the course of hardness measurements.

**Figure 12:**
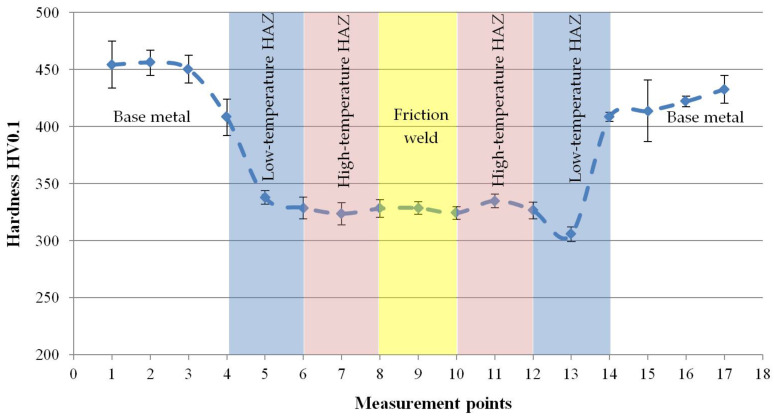
Hardness distribution in the friction-welded joint made of 316L steel after hydrostatic extrusion.

For the results to be statistically reliable, the measurements were made in four measurement lines. However, due to the small width of the individual zones of the joint, each of the measurement lines was divided into two separate lines in order to avoid the impact of the prints with each other. The course of hardness measurements (for 2 measuring lines) is shown in Figure 11. Figure 12 shows the obtained hardness distribution with the marked standard deviation.

The results of the hardness measurements showed that the change in the microstructure caused by the friction welding process (including the shape and orientation of the grains and the disorientation angle of the boundaries between them) reduced the hardness. Compared to the base material with hardness of approx. 430 HV0.1, the remaining part of the friction joint has a hardness of approx. 330 HV0.1 and small standard deviation bars indicate a more homogeneous nature of their microstructure.

## 4. Discussion and Conclusions

### 4.1. Base Material

Performing hydrostatic extrusion (in one pass) of 316L steel resulted in obtaining rods with high mechanical properties (Table 1). As a result of subjecting the material to high deformation, it is characterized by a limited possibility for further plastic processing and an increased sensitivity to heat [43]. Less thermal stability is caused by the strong deformation and grain refinement, the accumulation of additional energy in the material and limitation of the mobility of grain boundaries [44].

Microscopic tests have shown that in the case of 316L steel, the hydrostatic extrusion process causes a strong deformation of the equiaxial grains and the obtaining of a band microstructure. In the case of other materials subjected to this process, such microstructure was not obtained. The HE process caused such a strong deformation of the grains in 316L steel that it was not possible to fully determine their size even during TEM research. Nevertheless, it was observed that the width of individual bands is less than 500 nm, the clear boundaries between them are the slip planes, and inside the strands, areas with different dislocation density and sub-grains can be distinguished. The EBSD studies showed that HAGBs accounted for 72% of the base material, while among grain boundaries with a misorientation angle <15° as much as 22% accounted for the misorientation angle 3°. The average grain size was 1.7 µm, with 25.5% of grains below 1 µm. Numerous equiaxial grains with an area below 100 nm were observed around the deformed grains. The base material has a hardness of 430 HV0.1, but it is also characterized by considerable heterogeneity, as evidenced by the values of the standard deviation marked in the chart.

### 4.2. Heat-Affected Zone

In the HAZ, the dual nature of the microstructure was observed. The formation of two microstructures is primarily caused by a different amount of heat supplied to a given area during the friction phase of the joining process, therefore an additional division was made into low-temperature and high-temperature HAZ. In the literature [45] occur other divisions of the HAZ friction joint, e.g., the author divided the joint into the following zones: contact zone, fully plasticized zone, partly deformed zone, undeformed zone, thermomechanically treated [46].

In the case of the low-temperature heat-affected zone, bending of the base material strands was observed. The deformation of the bands from the horizontal to the vertical direction is primarily caused by the second stage of the friction welding process—the upsetting phase, in which the set pressure (upsetting pressure) increases, causing the plasticized material to be squeezed out and a flash formed.

The supplied heat allowed for deformation of the material in this zone, but also resulted in the loss of the effect of the lamellar microstructure observed during the TEM-investigation of the base material. Among the highly deformed microstructure of full dislocation tangles, it was possible to observe randomly spaced sharp boundaries of partially recrystallized grains. In the case of a high-temperature HAZ, the microstructure is still streaked—strands arranged along the weld. During SEM tests it was observed that they are cut by grain boundaries. TEM investigation reveals partially disappearance of sub-grains. The EBSD studies confirmed the presence of recrystallized grains among the strongly deformed (elongated) grains. The low-temperature HAZ was characterized by the highest average grain size of 5.8 µm, with a grain size share below 1 µm at the same level of 15% as for the high-temperature HAZ, with an average grain size of 2.7 µm. In both HAZ regions, the average grain boundary misorientation angle was below 30°, and the only zone in the friction joint with a comparable proportion of LAGBs and HAGBs boundaries is the low-temperature HAZ. However, in the entire HAZ, the share of boundaries with a misorientation angle of 3° remained above 20%. According to Humphrey’s theory on the influence of the nature of grain boundaries, LAGB subgrain structures are inherently unstable, and structures with medium and high misorientation angles may show greater stability [47]. Therefore, the nature of the grain boundaries also influences the thermal stability of UFG materials.

Regardless of the nature of changes in the observed microstructure, the heat generated in the friction welding process softened the material. In the HAZ zone, the hardness decreased by an average of 100 HV0.1. However, the HAZ zone has a more homogeneous microstructure than the base material (lower standard deviation bars of mean hardness value).

### 4.3. Friction Weld

As a result of the mutual friction of the faces of the welded bars and the generated heat, the friction weld has a completely different microstructure from the other areas of the friction-welded joints. The occurrence of dynamic recrystallization and mutual shear of the newly formed grains (due to the frictional moment) resulted in the formation of a homogeneous, fine-grained but also highly damaged (numerous dislocations inside and at the grain boundaries) microstructure. The average grain size was 2 µm, and the grain size distribution assumed the shape of the Gaussian distribution, where 47% of the grains have a surface area of 1–2 µm. The mean angle of misorientation was 32.5°, the value results, among others, from a large share of HAGBs at the level of 70% and a reduced to 16% share of grain boundaries with a misorientation angle of 3°. Despite the fact that such a microstructure was obtained, the friction weld did not have a different average hardness than the HAZ.

### 4.4. Micro Discontinuities of the Friction Weld Joint

During the SEM-investigation, micro discontinuities of a different nature were observed in individual zones of the friction joint, but it is difficult to clearly define the cause of their occurrence.

According to Erbel [48], in the case of materials subjected to the SPD process, the dislocation movement (as the basic mechanism of plastic deformation) does not disturb the integrity of the material. Only the hindrance of the dislocation movement by, for example, some grain boundaries or foreign inclusions create the risk of material discontinuity. Tests carried out by Erbel, consisting in twisting (in a special device) with arbitrarily large deformation of aluminum (99.5%), Armco iron and M63 brass confirmed that applying a sufficiently high 3-axis uniform compression prevents the formation of dislocation gaps inside the grains. On the other hand, discontinuities occurred in impurities clustered at the grain boundaries or crystals of the less plastic phase (in the case of M63).

Loss of cohesion of plastic materials, deformed with the participation of hydrostatic pressure (according to Erbel) takes place in the following stages: 1. It begins with cracking (brittle nature) of less plastic components, with relatively small deformations. 2. The spread of discontinuities is related to the sliding along the grain boundaries, taking place at much greater deformations. The less plastic components, without deforming, are an obstacle to displacement until they crumble. In this way, fractures of relatively large dimensions are formed along the grain boundaries, corresponding to the grain dimensions of the less plastic components.

At large deformations, when the texture that creates the grain boundaries coincide with the operating direction τ_max_ (as in torsion or hydrostatic extrusion), the resulting gap systems will be in parallel planes.

Similar material discontinuities were observed by Klassek et al. [49] in 316LVM steel after hydrostatic extrusion. During the tests of the charge material, despite the high purity of the steel, multi-phase inclusions, i.e., Al_2_O_3_, MnS and other Si-rich inclusions, were demonstrated. The process of hydrostatic extrusion (with ɛ_kum_ = 1.84) caused a change in the shape of inclusions—they were elongated in the extrusion direction and decreased in the cross-section.

According to the theory and results quoted, the application of the friction welding process caused further deformation of the micro discontinuities, but their final shape in a given area of the friction-welded joint depended on the conditions prevailing in this area. Unambiguous determination of the causes of the observed micro discontinuities requires additional research.

Conducting the process of high-speed friction welding of 316L steel after hydrostatic extrusion with a short friction time (60 ms) and rotational speed of 8000 rpm caused the release of heat, which initiated the process of recrystallization of single grains in heat-affected zone. The additional occurrence of strong plastic deformations (in HAZ) during flash formation and internal friction (in the friction weld and high-temperature HAZ) contributed to the formation of a highly deformed microstructure with numerous sub-grains.

A number of phenomena determining the properties of the joint, but interacting in the opposite way, have been observed. The friction-welded joint is characterized by a gradient of many properties, including structure directivity, degree of recrystallization, average grain size, and hardness.

The sub-grain structure significantly hinders the absolute measurement of grain size. And the obtained results are also relative in nature related to the magnification used for the observation.

The zones with a microstructure other than the base material were characterized by lower hardness. However, regardless of the nature of the changes in the microstructure, the level of hardness reduction in all zones was similar with a mild course of changes.

Due to the complexity of the microstructure and its multifactorial impact on the properties of the friction-welded joint, strength should be the criterion for assessing the properties of the joint.

## Figures and Tables

**Table 1:** Chemical composition (wt.%) of the examined stainless steel (manufacturer specification) and mechanical properties before and after HE.

	C	Si	Mn	P	S	N	Cr	Mo	Ni	Cu	Co
316L	0.017	0.36	1.82	0.30	0.026	0.077	16.88	2.04	10.14	0.38	0.10
		**Ultimate** **Tensile Strength**			**Yield** **Stress**		**Elongation** **to Fracture**			**Hardness**	
		**UTS (MPa)**			**YS (MPa)**		**εf (%)**			**HV0.2**	
316L		610			285		65			205	
316L after HE		1250			1180		11.9			355	
UFG 316L after HSFW		1080			1017		0.63			‒	

**Table 2:** Parameters of high-speed friction welding of 316L steel after HE.

Rotational speed set in the friction phase	8000 [rpm]
Friction phase duration	60 [ms]
Pressure on the front of the samples in the friction phase	255 [MPa]
Pressure on joint surface of the samples in the upset phase	318 [MPa]

## Data Availability

Experimental methods and results are available from the authors.
